# EPDR1, Which Is Negatively Regulated by miR-429, Suppresses Epithelial Ovarian Cancer Progression *via* PI3K/AKT Signaling Pathway

**DOI:** 10.3389/fonc.2021.751567

**Published:** 2021-12-23

**Authors:** Zhendan Zhao, Zhiling Wang, Pengling Wang, Shujie Liu, Yingwei Li, Xingsheng Yang

**Affiliations:** ^1^ Department of Gynecology and Obstetrics, Qilu Hospital of Shandong University, Jinan, China; ^2^ Laboratory of Basic Medical Sciences, Qilu Hospital of Shandong University, Jinan, China; ^3^ Department of Obstetrics and Gynecology, Zibo Spring Hospital Co., Ltd., Zibo, China

**Keywords:** EPDR1, miR-429, PI3K/AKT, ovarian cancer, tumor suppressor

## Abstract

Epithelial ovarian cancer (EOC) is the main pathological type of ovarian cancer. In this study, we found that ependymin-related 1 (EPDR1) was remarkably downregulated in EOC tissues, and low EPDR1 expression was associated with International Federation of Gynecology and Obstetrics (FIGO) stage, metastasis, and poor prognosis. We confirmed that EPDR1 overexpression dramatically suppressed EOC cell proliferation, migration, and invasion *in vitro* and *in vivo*. Mechanistically, EPDR1 inhibited EOC tumorigenesis and progression, at least in part, through the repression of the PI3K (Phosphoinositide 3-kinase)/AKT (AKT Serine/Threonine Kinase 1) signaling pathway. Furthermore, the expression and function of EPDR1 were regulated by miR-429, as demonstrated by luciferase reporter assays and rescue experiments. In conclusion, our study validated that EPDR1, negatively regulated by miR-429, played an important role as a tumor-suppressor gene in EOC development *via* inhibition of the PI3K/AKT pathway. The miR-429/EPDR1 axis might provide novel therapeutic targets for individualized treatment of EOC patients in the future.

## Introduction

Ovarian cancer, one of the most common gynecological malignancies in women worldwide (1), has a high incidence and high mortality rate and is especially difficult to discover during the early stages. Epithelial ovarian cancer (EOC) accounts for >95% of the ovarian malignancies and is the leading cause of gynecologic cancer deaths with a 5-year survival of 35% (2). The prognosis of EOC patients is extremely poor mainly for the following reasons. On one hand, owing to the small size of the ovary, which is located deep in the pelvic cavity, patients may not show any symptoms or may only vaguely show symptoms when tumor cells invade or spread to other parts of the body (3). On the other hand, the majority of women with advanced ovarian cancer will relapse after first-line treatment with platinum-based chemotherapy (4), and recurrent ovarian cancer is considered incurable (5). Therefore, it is important to find new therapeutic strategies and new biomarkers to ameliorate the clinical diagnosis and prognosis of EOC patients.

Ependymin-related 1 (EPDR1) is a member of mammalian ependymin-related proteins (MERPs). EPDR1, which is also known as EPDR, UCC1, and MEPR1, shares a conserved key amino acid and primary structure with piscine ependymin, which is a type II transmembrane protein and plays a crucial role in cell adhesion (6, 7). Human EPDR1 gene was first identified in two colorectal cancer (CRC) cell lines in 2001 (8). Although the function of EPDR1 has not been completely characterized, accumulating evidence suggests that EPDR1 is associated with various human diseases, particularly the initiation and progression of various human cancers (9, 10). At present, EPDR1 has been reported to be differentially expressed in a variety of tumors and exerts an oncogenic role in colorectal cancer (11) and a tumor-suppressive role in breast cancer (12). However, the characterization of EPDR1 gene in EOC has not been reported.

In this study, based on database mining of The Cancer Genome Atlas (TCGA), Gene Expression Omnibus (GEO), and Clinical Proteomic Tumor Analysis Consortium (CPTAC), RT-PCR, Western blot, and immunohistochemistry (IHC) were further performed to verify that EPDR1 was expressed at a low level in EOC tissues compared to that in normal ovary. More importantly, EPDR1 was also demonstrated to be a predictor of prognosis in EOC patients. Overexpression of EPDR1 inhibited the proliferation, migration, and invasion of EOC cells both *in vitro* and *in vivo*. Furthermore, we confirmed that EPDR1 expression was correlated with PI3K/AKT pathway and epithelial–mesenchymal transition (EMT) through Western blot, RT-PCR, and other experiments. To elucidate the regulatory mechanism underlying the expression of EPDR1 in EOC development, we focused on miRNA regulation mechanism and finally demonstrated that EPDR1 was directly targeted and regulated by miR-429 through a luciferase reporter assay and a series of rescue experiments. In conclusion, our findings might provide a promising therapeutic strategy for targeting EPDR1 in EOC.

## Materials and Methods

### Patients and Tissue Specimens

A total of 184 human EOC tissues and 84 normal ovary tissues samples were collected from the Department of Pathology, Qilu Hospital, between January 2012 and December 2015. Histopathologic and clinical characteristics of patients are shown in **Supplementary Table S1**. None of these patients had received previous radiotherapy or chemotherapy. The clinical diagnosis, International Federation of Gynecology and Obstetrics (FIGO) stage, and subtype of these samples were confirmed by three independent pathologists. The study was approved by the Research Ethics Committee of Shandong University Qilu Hospital.

### Immunohistochemistry

Paraffin-embedded tissues were cut into 4-μm sections and transferred to glass slides. After the sections were dried, dewaxed, hydrated, and underwent microwave antigen repairing, 3% hydrogen peroxide was used to blocked endogenous peroxidase activity for 10 min at room temperature. Sections were blocked with normal goat serum for 30 min at 37°C. Next, the samples were incubated with primary antibody against EPDR1 at a dilution of 1:500 (Origene, TA349935) at 4°C overnight. The slides were washed three times in phosphate buffered saline (PBS) buffer, followed by incubation for 1 h at room temperature with anti-rabbit horseradish peroxidase (HRP)-linked secondary antibody working solution (Zhongshan Golden Bridge, SAP-9100). Finally, the sections were visualized with the 3,3'-diaminobenzidine (DAB) chromogen. The EOC tissues were observed under a light microscope and scored by three senior pathologists. The final score was calculated by multiplying the staining intensity score by the staining area score, with a range between 0 and 12. Cases with greater or equal to 6 and less than 6 score value of EPDR1 immunostaining were regarded as high expression and low expression, respectively.

### RNA Extraction and Real-Time PCR

Total RNA from all clinical specimens and cells were harvested by TRIzol (Invitrogen, CA, USA) according to the manufacturer’s recommendations. After the determination of RNA concentration, PrimeScript RT reagent Kit With gDNA Eraser (Toyobo, Japan) was used to reverse-transcribe total RNA. RT-PCR was conducted by the SYBR Green Real-time PCR Master Mix (Toyobo, Japan). Primer sequences are listed in **Supplementary Table S2**. Data in this study were analyzed by 2-ΔΔct with U6 small nuclear RNA (U6) and Glyceraldehyde-3-phosphate dehydrogenase (GADPH) as endogenous controls.

### Cell Lines

A2780, HEY, SKOV3, and HEK293T cells were obtained from the Key Laboratory of Gynecologic Oncology of Shandong Province, and all cell lines were authenticated and regularly tested for mycoplasma. All of these cell lines were cultured by high-glucose Dulbecco's Modified Eagle Medium (DMEM) (Gibco, USA) supplemented with 10% fetal bovine serum (FBS) (Biological Industries, USA) and 1% penicillin/streptomycin (Hyclone SV30010). Cell lines were transfected with siRNAs, mimics, or inhibitors of miRNA by Lipo8000 Transfection Reagent (Beyotime Biotechnology, China). siRNA, mimics, and inhibitors were synthesized by GenePharma (China) (sequences are shown in **Supplementary Table S3**). After 48–72-h transfection, cells were collected and lysed to examine the transfection efficiency. EPDR1 overexpression lentivirus was purchased from Shanghai Jikai Gene Chemical Technology Co., Ltd. A2780 and HEY stable cell lines overexpressing EPDR1 were infected with lentivirus [multiplicity of infection (MOI): 50 and 10, respectively] and selected with 0.4 mg/ml and 0.8 mg/mg puromycin for about 1 week.

### Cell Proliferation Assays

The cell proliferation ability was determined using Cell Counting Kit-8 (CCK8, Dojindo, Japan). A2780 and HEY, which were transfected with EPDR1 lentiviral plasmid or siRNA, were seeded into 96-well plates (1,000 cells per well). At the harvest time, 10 µl of CCK8 was added into each well, and after 1-h incubation, cellular viability was determined by measuring the absorbance of the converted dye at 450 nm. For colony formation assay, cells were inoculated into six-well plates with 1,000 cells per well. After 2 weeks, the colonies were then fixed with 4% formaldehyde and stained with 0.5% crystal violet. Photos of stained colonies were taken and counted with ImageJ software. 5-ethynyl-2'-deoxyuridine (EdU) staining was performed to evaluate DNA synthesis in proliferating cells with an EdU assay kit (Ribobio, Guangzhou, China) following the manufacturer’s instructions. Images were photographed with a microscope (Olympus, Tokyo, Japan) and analyzed by ImageJ software. The ratio of EdU-stained cells to Hoechst-stained cells was used to evaluate proliferation.

### Wound Healing and Transwell Invasion Assay

A2780, HEY, and SKOV3 cells were seeded in a six-well plate and allowed to become confluent up to 100% until the next day. Next, a 200-µl pipette tip was used to scratch a straight line in the cell monolayer and replaced with serum-free medium. Images were captured by bright-field microscopy at time points 0 and 24 h. Photos were analyzed by ImageJ software to calculate the area of the scratch and represented as the percentage of wound closure. Cell invasion assays were performed in transwell chambers (BD Falcon, USA) precoated with Matrigel (BD Biosciences, USA). A2780, HEY, and SKOV3 cells were seeded at 100,000 cells/well on Matrigel-coated chambers with serum-free DMEM. The lower chamber was filled with 600 μl DMEM containing 20% FBS as chemoattractants. After 16 h, the invading cells on the underside of the transwell were fixed with 4% formaldehyde and stained with crystal violet. Five visual fields were randomly selected for each chamber.

### Western Blot

A total of protein from tissues and cells were extracted with radioimmunoprecipitation assay (RIPA) lysate buffer (Beyotime, Shanghai, China). After being lysed by sonication, the lysates were obtained by centrifugation at 13,000 rpm for 30 min. Then, the protein concentration was determined with bicinchoninic acid (BCA) assay kit (Beyotime, Shanghai, China). Protein of each sample was loaded and separated by electrophoresis with sodium dodecyl sulphate–polyacrylamide gel electrophoresis (SDS-PAGE) and then transferred to polyvinylidene difluoride (PVDF) membranes. Here, 12% SDS-PAGE was used to verify the protein whose molecular weight was less than 50 kDa, and other proteins were resolved by 10% SDS-PAGE gel. After being blocked for 2 h at room temperature with 5% skimmed milk in Tris Buffered Saline with Tween (TBST), the membranes were incubated with primary antibodies against EPDR1 (Origene, #TA349935), β-actin (Cell Signaling Technology, #3700), E-cadherin (Cell Signaling Technology, #14472), N-cadherin (Cell Signaling Technology, #13116), vimentin (Abcam, #ab92547), PI3K (Cell Signaling Technology, #4249), AKT (Cell Signaling Technology, 34691), P-AKT (Cell Signaling Technology, #4046), mammalian target of rapamycin (mTOR) (Cell Signaling Technology, #2983S), and P-mTOR (Cell Signaling Technology, #5536S) at 4°C overnight. The membranes were washed three times with TBST and incubated with a second antibody at a dilution of 1:5,000 (Beyotime, A0208) for 1 h at room temperature. Finally, the membranes were washed and visualized using chemiluminescence detection reagents (Thermo Scientific, A38555). Here, β-actin was used as a loading control to ensure equal loading. Protein expression levels were quantified using ImageJ software.

### Immunofluorescence Staining

A2780 and HEY, which were transfected with EPDR1 lentiviral plasmid or vector, were seeded into a 96-well plate at a density of 50,000 cells per well. After the cells adhered to the wall, cells were fixed with 4% formaldehyde and permeabilized with 0.1% Triton X-100. After being blocked with 10% normal goat serum for 30 min, cells were incubated with primary antibody against N-cadherin at a dilution of 1:200 (Cell Signaling Technology, #13116) and E-cadherin at a dilution of 1:200 (Cell Signaling Technology, #14472) at 4°C overnight. After being washed with PBS three times for 15 min, the cells were incubated with the respective fluorophore-conjugated secondary antibodies for 1 h at room temperature. Cell nuclei were stained with 4',6- diamidino-2-phenylindole (DAPI) (Boster Biological Technology Co. Ltd., Wuhan, China) for 10 min. Pictures were taken by a fluorescent microscope.

### Luciferase Activity Assay

HEK293T and HEY cells were seeded in a 24-well plate (30,000 cells/well). After being transfected with the indicated plasmid and miRNA mimics, cells were harvested and subjected to luciferase reporter assay using the dual luciferase assay kit (Beyotime, Shanghai, China). The relative luciferase activity was calculated by dividing the firefly luciferase activity by the Renilla luciferase activity.

### Transcriptome Sequencing and Bioinformatic Analysis

Genome-wide transcriptional sequencing was conducted by Sinotech Genomics Co., Ltd. (Shanghai, China). Transcriptome sequencing (NEB, USA) was used to identify mRNA transcripts with differential expression between EPDR1 overexpression A2780 cells and control A2780 cells. The genes with Log fold change (LogFC) >1.2 were thought to be differential genes. RNA sequencing data have been deposited to GEO database (GEO accession: GSE188918).

The gene expression data of EOC patients from TCGA and The Genotype-Tissue Expression (GTEx) were downloaded from UCSC Xena Browser (https://xenabrowser.net/). The protein expression profile of EOC patients was downloaded from CPTAC (https://cptac-data-portal.georgetown.edu/). The gene expression data of EOC patients from GEO datasets were obtained using GSE 14407 and GSE 18520 (https://www.ncbi.nlm.nih.gov/gds). Gene set enrichment analysis (GSEA) was performed using GSEA software (http://software.broadinstitute.org/gsea/index.jsp).

### Animal Experiment

All the animal experiment protocols were approved by the Medical Ethics Committee of Shandong University. BALB/c nude mice (immunodeficient, female, 4–6 weeks of age) were housed under standard conditions. Mice were randomly grouped for tumor cell injection. All analyses of animal experiments were performed by a blinded observer without knowledge of the experimental group. For subcutaneous xenograft experiments (n = 5), single-cell suspensions of HEY cell lines were prepared in PBS, and 5 million cells per 200-µl volume were injected in one subcutaneous site per mouse. Tumor volumes were measured with Vernier calipers every 5 days. The tumor volume was calculated by the formula: tumor volume = length × (width)2/2. After 4 weeks, the mice were sacrificed and the tumors were removed.

For peritoneal metastatic model, luciferase-labeled tumor cells (1 × 10^6^) in 1 ml PBS were intraperitoneally injected to the nude mice. HEY-Luciferase cells were acquired through transfection of lentivirus overexpressing the luciferase reporter gene into HEY cells. Luciferase reporter gene overexpression lentivirus was purchased from Shanghai Jikai Gene Chemical Technology Co., Ltd. After intraperitoneal administration of luciferase substrate (150 mg/kg; Biosynth International, Naperville, IL, USA), the anesthetized mice were imaged using an *in vivo* imaging system (PerkinElmer, Waltham, MA, USA) every 5 days.

### Statistical Analysis

All statistical analyses and graphical figures were done by GraphPad Prism v7 for Windows and R software v3.6.1. All experiments were repeated independently at least three times with at least three replicates. An unpaired t-test was used when comparing the different expressions of EPDR1 and miR-429 between normal tissue and tumor tissue and the abilities of proliferation, metastasis, and invasion between control group and EPDR1 overexpression group. One-way ANOVA was used for the comparison of multiple groups in rescue experiments and luciferase reporter experiments and when comparing the EPDR1 expression and abilities of proliferation, metastasis, and invasion between control group and two si-EPDR1 groups. Survival analysis was performed using Kaplan–Meier (K-M). The Pearson’s chi-square test was used as a test of significance for comparison of clinicopathological features. Correlation between EPDR1 and miR-429 expression was assessed by the use of Spearman’s correlation. Data are presented as bars with whiskers, showing means and standard deviation (SD). Also, error bars in bar graphs indicate SD.

## Results

### EPDR1 Expression Is Frequently Downregulated in EOC Tissues and Predicts Overall Survival Time in Epithelial Ovarian Cancer Patients

**Figure 1:**
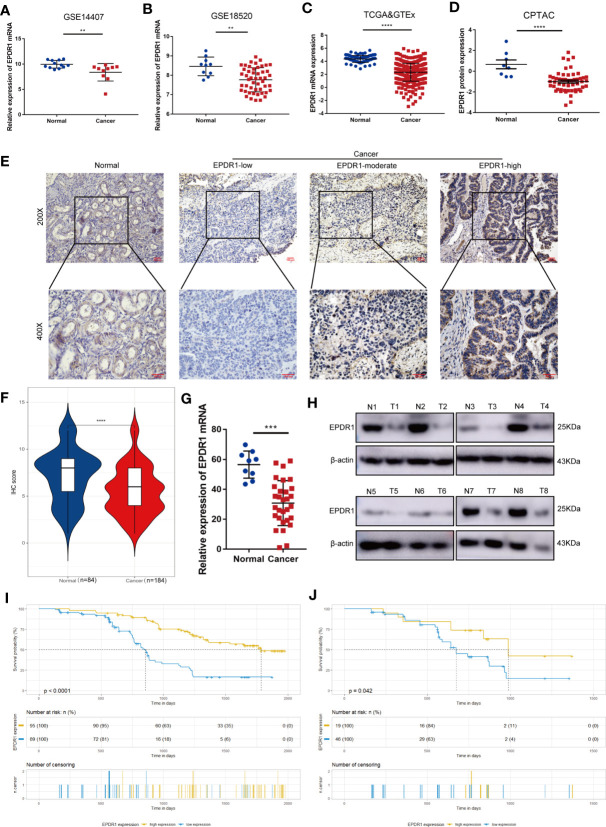
Expression and prognostic value of EPDR1 in EOC. **(A–D)** Expression analysis of EPDR1 in EOC and non-tumor tissues in GEO [GSE14407 **(A)** and GSE18520 **(B)**], TCGA, and GTEx datasets **(C, D)** and CPTAC datasets. Unpaired t test was conducted. Data are presented as mean ± SD. **(E**, **F)** IHC analysis **(E)** and score **(F)** of EPDR1 expression in EOC tissues and normal tissue in ×200 magnification (scale bar, 100 μm) and ×400 magnification (scale bar, 100 μm). Unpaired t test was conducted. Data are presented as mean ± SD. **(G, H)** RT-PCR and Western blot showing the differences of EPDR1 mRNA and protein levels in EOC tissues and normal tissues. N and T represent normal and tumor tissues, respectively. Unpaired t test was conducted. Data are presented as mean ± SD. **(I, J)** K-M survival curve analysis showing the correlation between the EPDR1 IHC score and OS in EOC patients **(I)** and patients in FIGO III–IV stage **(J)** **P < 0.01, ***P < 0.001, ****P < 0.0001.

Firstly, we used bioinformatic methods to explore the expression of EPDR1 in EOC. We analyzed GEO database (GES14407, GSE18520), TCGA, GTEx, and CPTAC database. As a result, we found that the expression of EPDR1 mRNA and protein was downregulated in EOC tissues compared to fallopian tube tissues and normal ovary tissues (**Figures 1A–D**). In order to validate these bioinformatic analysis results, EPDR1 mRNA level was assessed in EOC tissues and normal ovary tissues by RT-PCR. We also performed IHC and Western blot to explore the expression of EPDR1 protein between EOC tissues and normal ovary tissues. Consistent with the results from databases shown above, the expressions of EPDR1 mRNA and protein were significantly decreased in EOC tissues in contrast to normal tissues (**Figures 1E–H**).

We then analyzed the relationship between the clinicopathological parameters and EPDR1 expression in EOC patients. The results showed that 89 (48.4%) and 95 (51.6%) patients had low and high EPDR1 expression, respectively (**Table 1**). Meanwhile, EPDR1 low expression was significantly related to FIGO stage (P = 0.0021, **Table 1**), lymph node status (P = 0.0041, **Table 1**), and distant metastasis (P = 0.002, **Table 1**) but not to age, tumor size, or histological type (**Table 1**). Furthermore, K-M survival analysis indicated that patients with lower EPDR1 expression appeared to have a shorter overall survival (OS) (**Figure 1I**). This finding also coincided with the results from K-M plotter database (**Supplementary Figure S1A**). Remarkably, FIGO stage was one of the most critical independent risk factors for a poor prognosis in EOC (13). Then, we further probed the differential prognosis between EOC patients with high and low EPDR1 expression in a poor-prognosis subgroup, FIGO III–IV stage disease. Interestingly, in this subgroup, the patients with high expression levels of EPDR1 could also achieve longer OS than that in the low-expression cohort (**Figure 1J**).

**Table 1:** Correlation between EPDR1 expression and clinicopathological features in EOC.

Factors	Sample	EPDR1 expression		P value^a^
		Low expression	High expression	
Age				0.5515
<50	106 (57.6)	49	57	
≥50	78 (42.4)	40	38	
FIGO stage				0.0021
I–II	90 (64.7)	33	57	
III–IV	94 (35.3)	56	38	
Lymph node				0.0041
N0	136 (73.9)	57	79	
N1	48 (26.1)	32	16	
Distant metastasis				0.002
M0	163 (88.6)	72	91	
M1	21 (11.4)	17	4	
Tumor size				0.055
<5 cm	43 (23.3)	15	28	
≥5 cm	141 (76.6)	74	67	
Histological type				0.5575
Serous	154 (83.7)	76	78	
Others	30 (16.3)	13	17	

FIGO, International Federation of Obstetrics and Gynecology.

a The Pearson chi-square test was used.

### EPDR1 Inhibits EOC Cell Proliferation, Migration, and Invasion *In Vitro*

**Figure 2:**
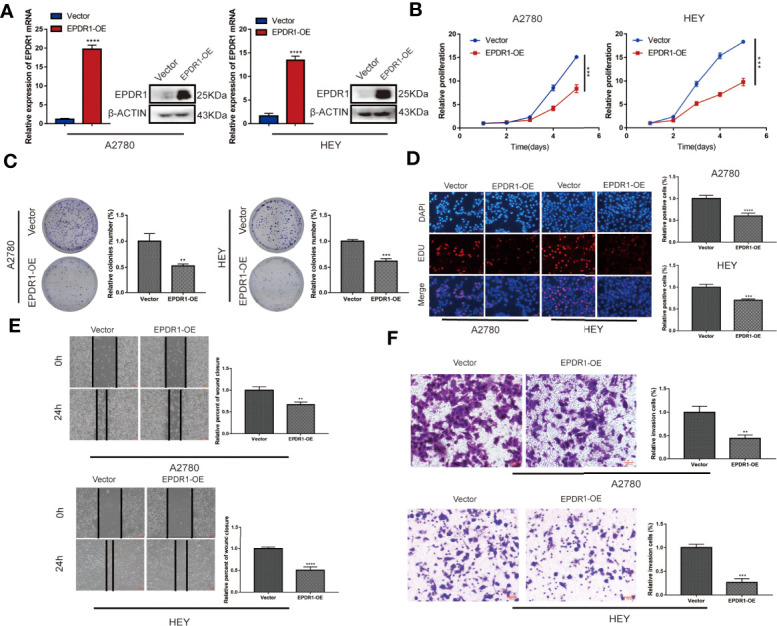
Overexpression of EPDR1 inhibits EOC cell proliferation and metastasis *in vitro*. **(A)** RT-PCR and Western blot were performed to detect the overexpression efficiency in A2780 and HEY. The amount of loading protein was 15 μg. Unpaired t test was conducted. Data are presented as mean ± SD. **(B–D)** Cell proliferation and DNA synthesis were analyzed by CCK-8 assay, colony formation assay, and EdU staining, respectively. Unpaired t test was conducted. Data are presented as mean ± SD. **(E)** A scratch assay was performed to examine the migration ability of A2780 and HEY cells after EPDR1 overexpression (magnification, ×40; scale bar, 100 μm). Unpaired t test was conducted. Data are presented as mean ± SD. **(F)** Transwell invasion experiment to detect the changes of invasion ability between the overexpression group and the control group (magnification, ×200; scale bar, 100 μm). Unpaired t test was conducted. Data are presented as mean ± SD. **P < 0.01, ***P < 0.001, ****P < 0.0001.

**Figure 3:**
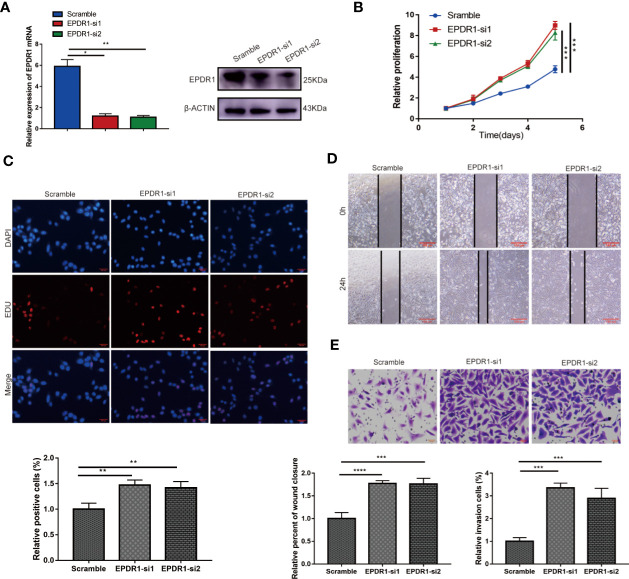
Knocking down EPDR1 expression promotes SKOV3 cell proliferation, migration, and invasion. **(A)** EPDR1 knockdown efficiency confirmed by RT-PCR and Western blot. The amount of loading protein was 30 μg. Results were analyzed with one-way ANOVA. Data are presented as mean ± SD. **(B, C)** Abilities of cell proliferation and DNA synthesis were determined by CCK-8 assay and EdU staining (magnification, ×200; scale bar, 100 μm). Results were analyzed with one-way ANOVA. Data are presented as mean ± SD. **(D, E)** Wound-healing assay (magnification, ×40; scale bar, 100 μm) and transwell invasion experiment (magnification, ×200; scale bars, 100 μm) were conducted to evaluate the migration and invasion capability between si-EPDR1 groups and control group in SKOV3 cells. Results were analyzed with one-way ANOVA. Data are presented as mean ± SD. *P < 0.05, **P < 0.01, ***P < 0.001, ****P < 0.0001.

We first detected the expression of EPDR1 in various ovarian cancer cell lines. Among these cell lines, SKOV3 cells showed the highest EPDR1 expression, while A2780 and HEY cells exhibited low EPDR1 expression (**Supplementary Figures S1B, C**). To further explore the role of EPDR1 in EOC development and progression, we selected A2780 and HEY with stable EPDR1 upregulation *via* transfection with lentiviral plasmids and SKOV3 with EPDR1 downregulation by transfection of EPDR1 small interfering RNA (siRNA). Overexpression and knockdown of EPDR1 were verified by RT-PCR and Western blotting (**Figures 2A**, **3A**). Cell Count Kit 8 assay (CCK-8), colony formation, and EdU assays showed that EPDR1 overexpression suppressed the cell proliferation, colony formation, and DNA synthesis (**Figures 2B–D**). The capabilities of migration and invasion were accessed in terms of scratch assay and transwell, respectively. As shown in **Figures 2E, F**, the abilities of cell migration and invasion were significantly decreased after EPDR1 overexpression.

In addition, we found that the knockdown of EPDR1 promoted the proliferation potential of SKOV3 cells, as demonstrated by CCK-8 assay and EdU assay (**Figures 3B, C** **)**. The capabilities of migration and invasion were also enhanced in cells transfected with si-EPDR1 (**Figures 3D, E** **)**. The gain-of-function and the loss-of-function data revealed that EPDR1 suppressed EOC growth and invasion.

### EPDR1 Inhibits EOC Development *In Vivo*

**Figure 4:**
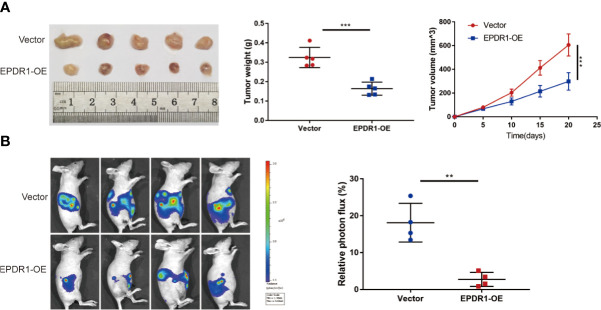
EPDR1 inhibits tumorigenesis and progression of ovarian cancer *in vivo*. **(A)** Representative images of tumor from EPDR1 overexpression group and control group after subcutaneous injection with HEY cells (n = 5 mice per group). The volumes and weights were lower for xenograft tumors with EPDR1 overexpression than that with vector. Unpaired t test was conducted. Data are presented as mean ± SD. **(B)** The relative luciferase activity and the representative photo flux in immunodeficient mice 4 weeks after intraperitoneal injection of luciferase-expressing HEY cells with EPDR1 overexpression or not. Unpaired t test was conducted. Data are presented as mean ± SD. **P < 0.01, ***P < 0.001.

To further address the role of EPDR1 in EOC progression, we generated subcutaneous xenograft tumor mouse models of EOC. HEY cells with stable EPDR1 overexpression or not were subcutaneously injected into nude mice (n = 5). As expected, overexpression of EPDR1 remarkedly reduced the tumor volume and weight (**Figure 4A**). Next, to access the effects of EPDR1 on EOC metastasis *in vivo*, we conducted intraperitoneal injection. Luciferase-expressing HEY cells were intraperitoneally injected into nude mice (n = 4). The results of living-imaging showed that EPDR1 overexpression caused a significant reduction in tumor burden (**Figure 4B**). Taken together, these findings supported the notion that EPDR1 played a role of tumor suppressor in EOC progression.

### EPDR1 Inhibits EOC Progression Through PI3K/AKT/mTOR Pathway

**Figure 5:**
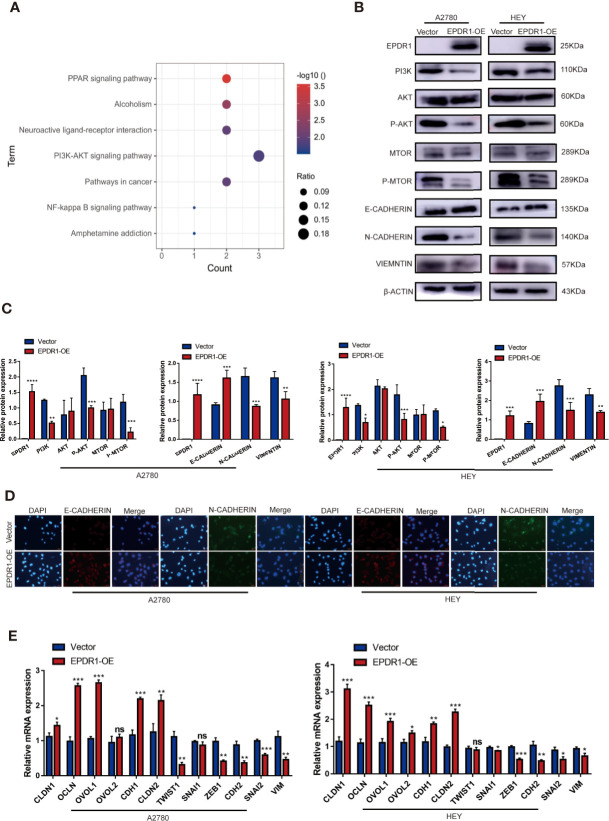
EPDR1 overexpression inhibits PI3K/AKT pathway and EMT progression. **(A)** KEGG pathway analysis was performed in EPDR1-overexpressing A2780 cells compared to negative control cells. **(B, C)** Different expression and analysis of PI3K/AKT pathway-related proteins and EMT-associated markers between EPDR1 overexpression group and control group. The amount of loading protein was 15 μg. Unpaired t test was conducted. Data are presented as mean ± SD. **(D)** Immunofluorescence staining showed that N-cadherin was downregulated and E-cadherin was upregulated after EPDR1 overexpression in A2780 and HEY (magnification, ×200; scale bar, 100 μm). **(E)** Mesenchymal-associated transcription factors (such as *ZEB1*, *CDH2*, *SNAI2*, *SNIA1*, *VIM*, and *TWIST1*) were downregulated, and epithelial-associated transcription factors (such as *CLDN1*, *OVOL2*, *CLDN2*, *OVOL1*, *OCLN*, and *CDH1*) were upregulated in EPDR1 overexpression group in A2780 and HEY by RT-PCR assay. Unpaired t test was conducted. Data are presented as mean ± SD. *P < 0.05, **P < 0.01, ***P < 0.001.

To gain insight into the molecular mechanism of EPDR1 in EOC progression, transcriptome sequencing was performed by using EPDR1-overexpressing A2780 cells and control cells. Then, we conducted comprehensive bioinformatic methods to analyze the transcriptome sequencing results. The results of Gene Ontology (GO) enrichment analysis exhibited a positive correlation between high EPDR1 expression and transmembrane receptor protein tyrosine kinase signaling pathway, regulation of nucleic acid-templated transcription, regulation of gene expression and differentiation, and many other functions (**Supplementary Figures S1D, E**). The Kyoto Encyclopedia of Genes and Genomes (KEGG) pathway enrichment analysis showed that EPDR1 was closely associated with PI3K/AKT signaling pathway (**Figure 5A**). We further confirmed the expression of PI3K/AKT pathway-related proteins (PI3K, AKT, P-AKT, mTOR, and P-mTOR). The results revealed that overexpressing EPDR1 suppressed the protein level of PI3K, P-AKT, and P-mTOR in both A2780 and HEY cell lines. Besides, EPDR1 also altered the expression of EMT-related key factors, including upregulation of E-cadherin, downregulation of N-cadherin and vimentin (**Figures 5B, C** **)**. Thereafter, we verified the differential expression of E-cadherin and N-cadherin by immunofluorescence (**Figure 5D**). In addition,12 markers in EMT were further validated by subsequent RT-PCR. As a result, the epithelial-associated transcription factor expression (such as CLDN1 and OCLN) was upregulated in EPDR1-overexpressing cells while mesenchymal-associated transcription factors were downregulated (**Figure 5E**).

### miR-429 Directly Targets EPDR1, Whose Expression Is Significantly Upregulated in EOC Patients

**Figure 6:**
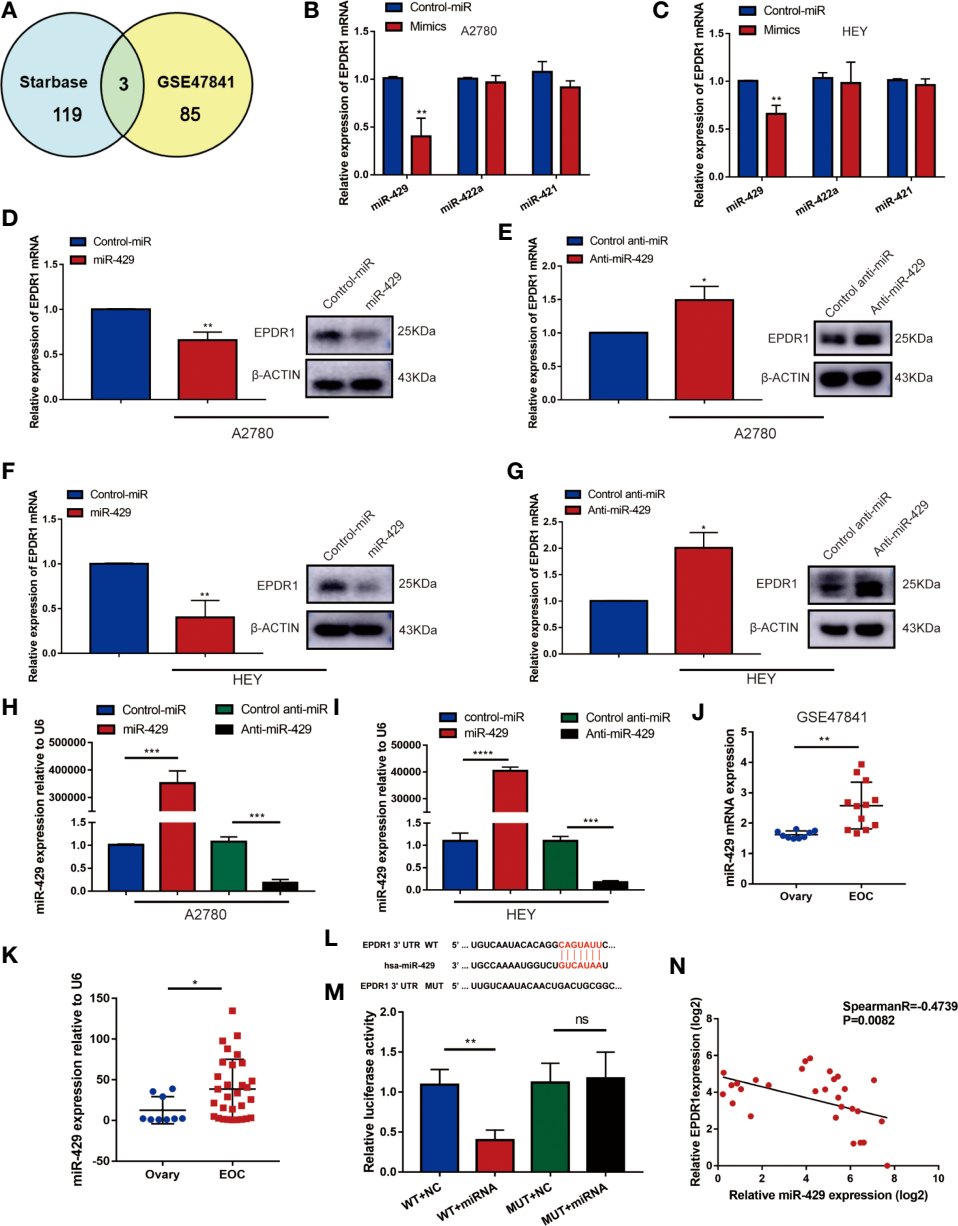
miR-419 directly targets EPDR1 whose expression is significantly upregulated in EOC patients. **(A)** Schematic illustration displaying the overlapped upstream miRNA of EPDR1 predicted by Starbase and GEO dataset (GSE47841). **(B, C)** The mRNA expression of EPDR1 was tested by RT-PCR in A2780 and HEY after transfection with mimics of top 3 candidate miRNAs. Unpaired t test was conducted. Data are presented as mean ± SD. **(D–G)** RT-PCR and Western blot were performed to detect the expression of EPDR1 after transfection with mimic and inhibitor of miR-429 in A2780 and HEY. The amount of loading protein was 50 μg. Unpaired t test was conducted. Data are presented as mean ± SD. **(H, I)** Transfection efficiencies of miR-429 mimic and inhibitor were evaluated by RT-PCR in A2780 and HEY. Results were analyzed with one-way ANOVA. Data are presented as mean ± SD. **(J)** The expression of miR-429 in EOC tissue sample and normal tissue sample from GSE47841 dataset. Unpaired t test was conducted. Data are presented as mean ± SD. **(K)** RT-PCR result showed that miR-429 was upregulated in EOC patients. Unpaired t test was conducted. Data are presented as mean ± SD. **(L)** The sequence of binding targets of miR-429 in WT or mutant EPDR1 3′-UTR. **(M)** After transfection with miR-NC or miR-429 in HEK293T cells, the relative luciferase activity of mutant or wild-type EPDR1 3′-UTR was detected. Results were analyzed with one-way ANOVA. Data are presented as mean ± SD. **(N)** The correlation between the expression levels of miR-429 and EPDR1 in EOC patients as determined by RT-PCR. Statistical analysis was performed using a Spearman correlation. *P < 0.05, **P < 0.01, ***P < 0.001.

To investigate the underlying molecular mechanism that was involved in the tumor suppressor role of EPDR1, we examined whether miRNAs could regulate EPDR1 levels in EOC patients. We screened potential miRNAs that could interact with EPDR1 based on the bioinformatic algorithms of Starbase (http://starbase.sysu.edu.cn/). A total of 119 miRNAs were predicted to bind to the 3′-untranslated region (UTR) of EPDR1. Next, we selected the intersections of candidate miRNAs from Starbase and miRNAs that were upregulated in dataset GSE47841 according to miRNA–mRNA regulation mechanism (**Figure 6A**). Finally, three miRNAs meeting these requirements were tested, including miR-429, miR-422a, and miR-421. The comprehensive list of the identified miRNAs is provided in **Supplementary Table S4**. RT-PCR was conducted to test the mRNA expression after being transfected by their mimics. We found that only miR-429 could significantly decrease EPDR1 expression in both A2780 and HEY cell lines (**Figures 6B, C** **)**. Furthermore, we performed Western blot to confirm that the protein level of EPDR1 was downregulated by miR-429. After being transfected by miR-429 inhibitor, the expression of EPDR1 was reversed (**Figures 6D–G**). The transfection efficiency was shown in **Figures 6H, I**. Next, to verify the expression of miR-429 in EOC patients, we fetched expression values of miR-429 in GSE47841. Besides, the relative expression level of miR-429 was also assessed by RT-PCR in EOC tissues and normal ovary tissues. Results showed that compared to normal tissue, miR-429 was overexpressed in EOC tissues (**Figures 6J, K** **)**.

Furthermore, to identify the relationship between miR-429 and the 3′-UTR of EPDR1, a luciferase reporter assay was performed. We cloned the sequence of EPDR1 3′-UTR flanked wild-type (WT) or mutant binding target of miR-429 into pmiRGLO vector, respectively (**Figure 6L**). We confirmed that a miR-429 mimic efficiently decreased the luciferase activity of an EPDR1-3′-UTR-WT reporter in HEK293T cells but not that of a 3′-UTR-mutated EPDR1 reporter (**Figure 6M**). This result was also verified in HEY cells (**Supplementary Figure S1F**). Moreover, the relative expression levels of miR-429 and EPDR1 in EOC samples and their relationship were further verified in EOC tissue samples by RT-PCR (**Figure 6N**; Spearman R = -0.4739, P = 0.0082). In conclusion, EPDR1 was a direct target of miR-429 in EOC.

### The Role of EPDR1 on Tumor Progression Is Regulated by miR-429

**Figure 7:**
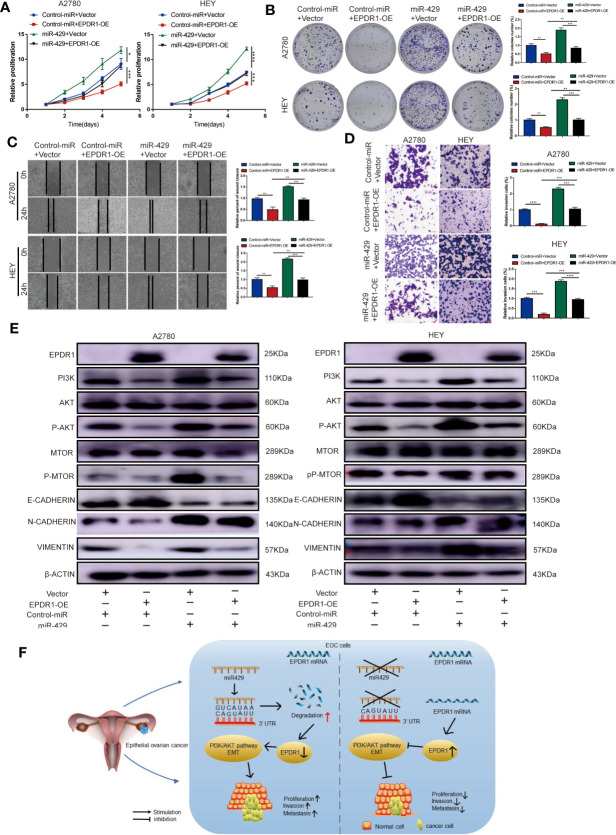
The functional roles of EPDR1 in EOC are regulated by miR-429 expression levels. **(A, B)** Proliferation of EOC cells after cotransfection with miR-429 mimic or NC mimic with either EPDR1 overexpression or not was analyzed by CCK-8 assay and colony formation assay. Results were analyzed with one-way ANOVA. Data are presented as mean ± SD. **(C, D)** Wound healing **(C)** and invasion assay **(D)** of the rescue experiment in A2780 and HEY cells (scale bar, 100 μm). Results were analyzed with one-way ANOVA. Data are presented as mean ± SD. **(E)** Western blot analysis of PI3K/AKT signaling pathway and EMT-related markers of rescue experiment in A2780 and HEY cells. The amount of loading protein was 15 μg. **(F)** A proposed mechanism scheme for EPDR1 regulated by miR-429 suppressed epithelial ovarian cancer proliferation and metastasis through the PI3K/AKT pathway. *P < 0.05, **P < 0.01, ***P < 0.001, ****P < 0.0001.

To further verify whether miR-429 was involved in EPDR1-mediated proliferation and metastasis effects in EOC cells, we conducted a rescue experiment. A2780 and HEY cells were co-transfected with miR-429 mimic or normal control (NC) mimic and EPDR1 overexpression lentiviral plasmid or vector. As shown in **Figures 7A–D**, cell line capabilities of proliferation, migration, and invasion were specially increased after upregulating miR-429, while the promoting effect could be partially reversed by EPDR1 overexpression. To further understand the potential mechanism of EPDR1-mediated effect by miR-429, we detected the key protein in PI3K/AKT signal pathway and EMT-related protein. As a result, PI3K/AKT pathway was significantly activated after miR-429 overexpression, which was rescued by co-transfection with EPDR1 lentiviral plasmid. Meanwhile, EMT process was promoted by miR-429, which could be partially blunted by EPDR1 (**Figure 7E**). In summary, these results demonstrate that the inhibition effect of EPDR1 on tumor progression is regulated by miR-429 through PI3K/AKT pathway (**Figure 7F**).

## Discussion

The mortality rate of ovarian cancer is the highest of the gynecological cancers, and the overall 5-year survival rate of EOC is below 50% (2). Hence, it is essential to identify the novel molecular targets and investigate the potential mechanism. In our study, we found that EPDR1 was downregulated in EOC tissue samples, and low EPDR1 was associated with tumor progression and poor prognosis in EOC patients by analysis of TCGA, GTEx, GEO and CPTAC dataset, RT-PCR, Western blot, and IHC. Furthermore, we confirmed that EPDR1 overexpression remarkedly suppressed EOC cell proliferation, migration, and invasion. Activation of PI3K/AKT signaling pathway was significantly inhibited after overexpression of EPDR1. In addition, EPDR1 was demonstrated to be the direct target of miR-429 and was regulated by miR-429 during EOC progression through PI3K/AKT pathway.

EPDR1, located on chromosome 7p14.1 (8), is conserved across species (14). The orthologs of EPDR1 in mouse and human are expressed in various normal tissues and in cancerous cell lines (15). Through sequence alignment, Gregorio-King et al. (16) found that EPDR1 had significant homology to the cytoplasmic domains of members of the protocadherin family of Type I transmembrane glycoproteins, which was postulated to function in cell adhesion and cellular signaling. In recent years, a growing number of studies have been performed to demonstrate the association of EPDR1 and cancers. In 2016, Riffo-Campos et al. reported that EPDR1 and its spliced isoforms are differentially expressed in human colorectal cancer cell lines. The expression of EPDR1 was underexpressed in the Caco2, RKO, and SW48 cell lines, while the HCT116, DLD1, and D-Mut1 cells overexpressed EPDR1 (9). Gimeno-Valiente et al. (11) found that upregulation of EPDR1 in DLD1 and HCT116 cell lines was related to human colorectal cancer stage, and knockdown of EPDR1 could inhibit cell proliferation, migration, invasiveness, and adhesion to type I collagen fibers. Similar results were observed in human bladder cancer (17). However, it has been proven that EPDR1 exhibited a lower expression level in breast cancer tissues compared to adjacent normal tissues and functioned as a tumor suppressor in breast cancer cells (12). The different roles of EPDR1 gene in diverse cancers could be attributed to the epigenetic silence due to DNA methylation (10). Consistent with the results observed in breast cancer, our data indicated that EPDR1 was downregulated in EOC tissues and low expression of EPDR1 was associated with poor prognosis. Furthermore, *in vitro* and *in vivo* functional studies confirmed that overexpression of EPDR1 inhibited cell proliferation, migration, and invasion. These results implied that EPDR1 played an important role in EOC progression.

Next, we focused on the underlying mechanism by which EPDR1 inhibits tumor progression. We conducted bioinformatic analysis based on transcriptome sequencing and TCGA dataset. EPDR1 expression was closely correlated with the expression of PI3K/AKT pathway. PI3K/AKT signaling pathway, which is one of the most frequently altered pathways in human cancer, plays a critical role in driving tumor initiation and progression (18). AKT could induce nuclear entry of Mdm2, which leads to inhibition of p53-regulated processes, resulting in increasing insensitivity to antiproliferative signals (19). AKT-mediated phosphorylation negatively regulated numerous proapoptotic factors, including Bad 28 and procaspase-9, thus increasing resistance to apoptosis of tumor cells (20). In addition, a previous study has reported that activation of PI3K/AKT signaling pathway could suppress GSK-3β and increase the level of Snail correspondingly, which led to the downregulation of E-cadherin and the subsequent EMT progression (21). Clinically, PI3K/AKT signaling is highly activated in ovarian cancer, and vast preclinical studies and clinical data suggested that inhibitors of PI3K/AKT pathway exerted an antitumor efficiency in the treatment of ovarian cancer (22). In the current study, we demonstrated that EPDR1 inhibited proliferation, invasion, and migration *via* suppressing the PI3K/AKT pathway. We also proved that EPDR1 attenuated the EMT process through a series of experiments. Our data were not sufficient to prove the interaction between EPDR1 and PI3K/AKT pathway. To probe the mechanism of interaction between EPDR1 and PI3K/AKT signaling pathway, the cBioPortal (http://www.cBioportal.org) was used to identify the genes co-expressed with EPDR1. We found that there was a significant inverse correlation between EPDR1 and SNCG. SNCG is also known as synuclein γ. The C-terminal tail region of SNCG was enriched with highly charged residues, which was reported to be able to make interaction with the residues of αC helix and β4 sheet of the AKT kinase domain, thus stimulating the activation of AKT (23). Meanwhile, SNCG could bind to transcription factor TWIST, and knockdown of SNCG inhibited TWIST-induced cell invasion and EMT (24). Therefore, we speculated that there was a negative regulatory relationship between EPDR1 and SNCG. EPDR1 inhibited PI3K/AKT pathway and EMT possibly through SNCG. Taken together, these results confirmed that EPDR1 played the role of a tumor suppressor gene in ovarian cancer through inhibiting the PI3K/AKT pathway and the process of EMT.

As mentioned above, in studies of the relationship between EPDR1 and tumors, EPDR1 played different roles in various tumors. To go further in the characterization of the regulatory mechanisms that are influencing EPDR1 expression phenotype, we assumed that EPDR1 was regulated by upstream miRNA. miRNA is a group of small endogenous noncoding single-strand RNAs (~21–25 nt), which negatively regulates gene expression at the posttranscriptional level (25). miRNA plays an important role in the detection, diagnosis, treatment, and prognosis in gynecological tumors (26–28). miRNA could serve as an oncogene and play an important role in tumorigenesis ang tumor development (29). In the present study, we confirmed the direct interaction between EPDR1 and miR-429. Previous studies have been reported that miR-429 participated in carcinogenesis. In breast cancer, oncogenic miR-429 was able to regulate hypoxia inducible factor-1α (HIF1α) pathway by directly targeting von-Hippel Lindau (VHL) mRNA, which caused increased proliferation and migration of breast cancer cells (30). Li et al. (31) found that miR-429 expression was upregulated in human colorectal cancer tissues, and high miR-429 expression was significantly associated with tumor size, lymph node metastasis, and poor prognosis. In addition, previous studies have proven that miR-429 unregulated in serous ovarian cancer and endometrial cancer (32, 33). In our study, we confirmed that miR-429 promoted cell proliferation and metastasis in EOC cells. Moreover, we identified that the roles of EPDR1 could be regulated by miR-429 expression levels in EOC by rescue experiments. After overexpressing EPDR1, the cancer-promoting effect of miR-429 was partially repressed, which suggested the presence of additional targets of miR-429 in EOC (34, 35). Large tumor suppressor kinase 2 (LAST2) was reported to inhibit the proliferation and invasion as well as dictate senescence in ovarian cancer. In colorectal cancer, LAST2 is the direct target for miR-429. Overexpression of LAST2 could downregulate the cancer-promoting effect (36). Thus, we presume that LAST2 is the other target of miR-429 in EOC. To summarize, our findings demonstrated that miR-429 was the direct upstream molecule of EPDR1 and regulated expression and functions of EPDR1. Thus, miR-429/EPDR1 may be a potential therapeutic target for the antitumor therapy in EOC and remains to be further explored.

We acknowledge that limitations exist in regard to this study. Firstly, the detailed molecular mechanism remains to be further elucidated such as interaction of PI3K/AKT and EMT signaling pathway inhibition induced by EPDR1 overexpression. Secondly, few studies investigated the role of EPDR1 in EOC progression, which limited our further investigation of the molecular mechanism. Our results lay the groundwork for further studies to explore downstream mechanisms of EPDR1 downregulation in EOC progression.

All in all, we demonstrated that EPDR1 was downregulated in EOC tissues, and low EPDR1 expression was correlated with tumor stage, lymph node and distant metastasis, and poor prognosis. This study is the first demonstration of the prognostic specificity of EPDR1 in EOC patients. Moreover, EPDR1 overexpression could significantly suppress the proliferation and metastasis of EOC cells, which was mediated through the PI3K/AKT pathway. Furthermore, miR-429 was verified as the upstream molecule that directly targeted EPDR1 and regulated the function of EPDR1 in EOC. The newly identified miR-429/EPDR1 axis may provide promising new therapeutic targets and prognostic biomarkers for patients with EOC.

## Data Availability Statement

The original contributions presented in the study are included in the article/**Supplementary Material**. Further inquiries can be directed to the corresponding author.

## Ethics Statement

The studies involving human participants were reviewed and approved by the Medical Ethics Committee of Shandong University. The patients/participants provided their written informed consent to participate in this study. The animal study was reviewed and approved by the Medical Ethics Committee of Shandong University.

## Author Contributions

XY and ZZ conceived and designed the experiments. ZZ, ZW, and SL performed the experiment work and analyzed the data. ZZ wrote the article. YL and PW completed the bioinformatic analysis. All authors contributed to the article and approved the submitted version.

## Funding

The study was financially supported by the National Natural Science Foundation of China (no. 81874105).

## Conflict of Interest

Author SL was employed by company Zibo Spring Hospital Co., Ltd.

The remaining authors declare that the research was conducted in the absence of any commercial or financial relationships that could be construed as a potential conflict of interest.

## Publisher’s Note

All claims expressed in this article are solely those of the authors and do not necessarily represent those of their affiliated organizations, or those of the publisher, the editors and the reviewers. Any product that may be evaluated in this article, or claim that may be made by its manufacturer, is not guaranteed or endorsed by the publisher.
