# Supplementary material for: EPDR1, Which Is Negatively Regulated by miR-429, Suppresses Epithelial Ovarian Cancer Progression via PI3K/AKT Signaling Pathway
Source: Front Oncol. 2021 Dec 23;11:751567. doi: 10.3389/fonc.2021.751567 (PMC8733570; doi:10.3389/fonc.2021.751567)
Supplement: Supplementary file 1 [file DataSheet_1.pdf]

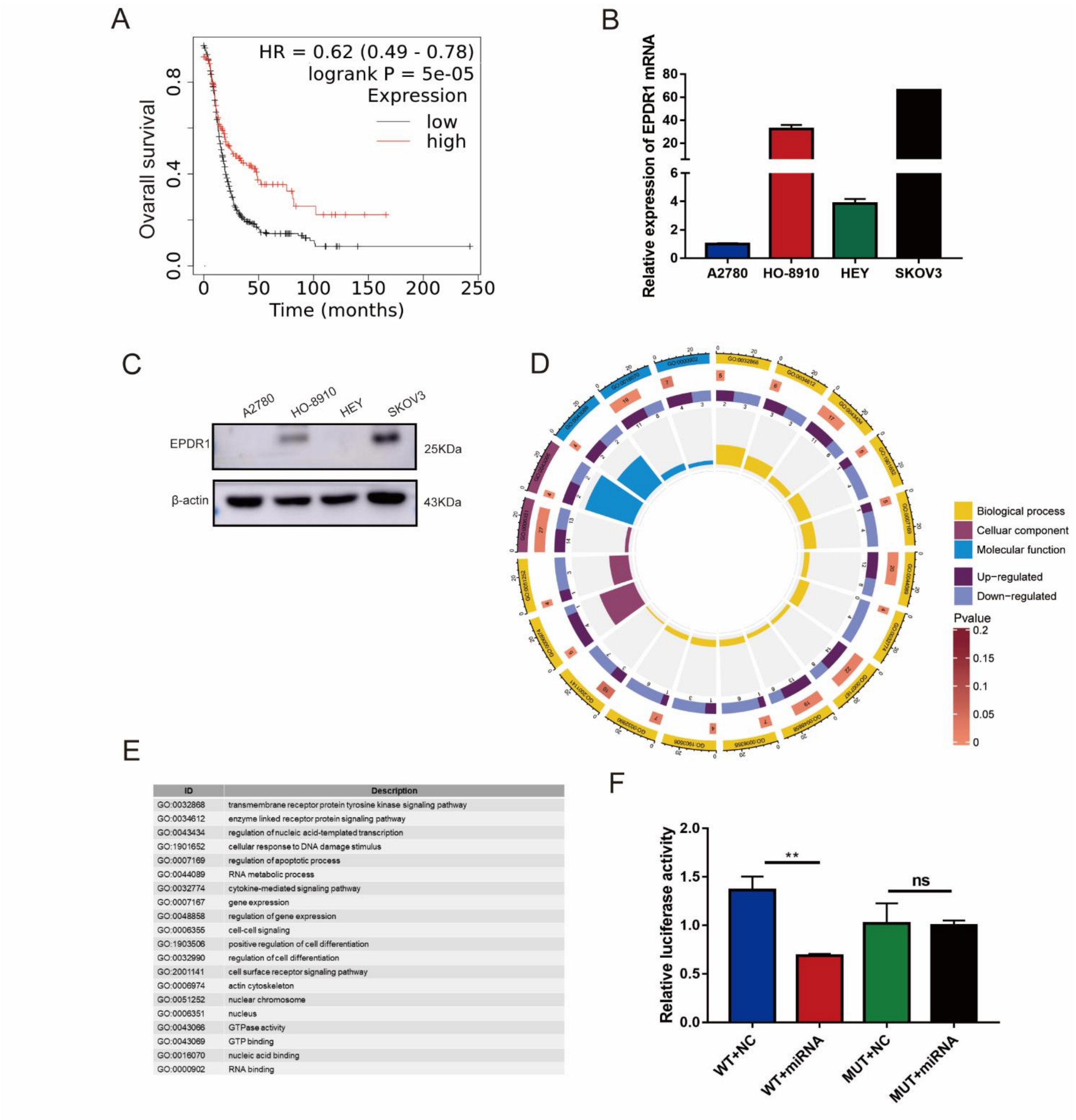

ID	Description
GO:0032868	transmembrane receptor protein tyrosine kinase signaling pathway
GO:0034612	enzyme linked receptor protein signaling pathway
GO:0043434	regulation of nucleic acid-templated transcription
GO:1901652	cellular response to DNA damage stimulus
GO:0007169	regulation of apoptotic process
GO:0044089	RNA metabolic process
GO:0032774	cytokine-mediated signaling pathway
GO:0007167	gene expression
GO:0048858	regulation of gene expression
GO:0006355	cell-cell signaling
GO:1903506	positive regulation of cell differentiation
GO:0032990	regulation of cell differentiation
GO:2001141	cell surface receptor signaling pathway
GO:0006974	actin cytoskeleton
GO:0051252	nuclear chromosome
GO:0006351	nucleus
GO:0043066	GTPase activity
GO:0043069	GTP binding
GO:0016070	nucleic acid binding
GO:0000902	RNA binding

Supplementary FigureS1. (A) Survival analysis of EPDR1 with ovarian cancer patients with high EPDR1 expression and low EPDR1 expression from K-M plotter. **(B, C)** Expression level of EPDR1 in different cell lines. (D,E) GO analysis in EPDR1 overexpressed A2780 cells compared to negative control cells. (F) Luciferase report assays for EPDR1 and miR429 in HEY cells. Results were analyzed with one-way ANOVA. Data are presented as mean±SD.
