# Supplementary material for: EPDR1, Which Is Negatively Regulated by miR-429, Suppresses Epithelial Ovarian Cancer Progression via PI3K/AKT Signaling Pathway
Source: Front Oncol. 2021 Dec 23;11:751567. doi: 10.3389/fonc.2021.751567 (PMC8733570; doi:10.3389/fonc.2021.751567)
Supplement: Supplementary file 2 [file Table_1.docx]

| **Supplementary TableS1. Clinical and histopathological characteristics of patients** | |
| --- | --- |
| **Characteristics** | **Cohort (n=184)**  **No. of patients** |
| Age  <50  ≥ 50 | 106 (57.6%)  78 (42.4) |
| FIGO stage |  |
| Ⅰ-Ⅱ | 90 (64.7%) |
| Ⅲ-Ⅳ | 94 (35.3%) |
| Lymph node |  |
| N0 | 136 (73.9%) |
| N1 | 48 (26.1%) |
| Distant metastasis |  |
| M0 | 163 (88.6%) |
| M1 | 21 (11.4%) |
| Histology type  Serous  Non-serous | 154 (83.7%)  30 (16.3%) |
|  |  |
