# Supplementary material for: EPDR1, Which Is Negatively Regulated by miR-429, Suppresses Epithelial Ovarian Cancer Progression via PI3K/AKT Signaling Pathway
Source: Front Oncol. 2021 Dec 23;11:751567. doi: 10.3389/fonc.2021.751567 (PMC8733570; doi:10.3389/fonc.2021.751567)
Supplement: Supplementary file 3 [file Table_2.docx]

| **Supplementary TableS2. RT-PCR primers of mRNA and miRNA** | |
| --- | --- |
| **mRNA** | **primer sequences** |
| Human EPDR1 Forward Primer | 5’- GCTGATCCCCTGCAAGAGATTATTT-3’ |
| Human EPDR1 Reverse Primer | 5’- CCCCGATGGAGTACTGGTCT-3’ |
| Human GADPH Forward Primer | 5’- GGAGCGAGATCCCTCCAAAAT-3’ |
| Human GADPH Reverse Primer | 5’- GGCTGTTGTCATACTTCTCATGG-3’ |
| Human CDH1 Forward Primer | 5’- GCTGGACCGAGAGAGTTTCC-3’ |
| Human CDH1 Reverse Primer | 5’- CGACGTTAGCCTCGTTCTCA-3’ |
| Human ZEB1 Forward Primer | 5’- ATGCAGCTGACTGTGAAGGT-3’ |
| Human ZEB1 Reverse Primer | 5’- GCCCTTCCTTTCCTGTGTCA-3’ |
| Human OVOL1 Forward Primer | 5’- CGGCTCCCGGCTTCAGTTA-3’ |
| Human OVOL1 Reverse Primer | 5’- CCCAGGCTGACTGGCAC-3’ |
| Human OVOL2 Forward Primer | 5’- CCATGCCCAAAGTCTTCCTG-3’ |
| Human OVOL2 Reverse Primer | 5’- GCCTAGGCCCACTGGGAT-3’ |
| Human CDH2 Forward Primer | 5’- AGGCTTCTGGTGAAATCGCA-3’ |
| Human CDH2 Reverse Primer | 5’- TGCAGTTGCTAAACTTCACATTG-3’ |
| Human VIM Forward Primer | 5’- GGGACCTCTACGAGGAGGAG-3’ |
| Human VIM Reverse Primer | 5’- TCCTCCTGCAATTTCTCCCG-3’ |
| Human SNAIL1 Forward Primer | 5’- CGAGTGGTTCTTCTGCGCTA-3’ |
| Human SNAIL1 Reverse Primer | 5’- CTGCTGGAAGGTAAACTCTGGA -3’ |
| Human SNAIL2 Forward Primer | 5’- ACTGGACACACATACAGTGATT-3’ |
| Human SNAIL2 Reverse Primer | 5’- ACTCACTCGCCCCAAAGATG-3’ |
| Human TWIST1 Forward Primer | 5’- GCCGGAGACCTAGATGTCATT-3’ |
| Human TWIST1 Reverse Primer | 5’- TTTTAAAAGTGCGCCCCACG-3’ |
| Human OCLN Forward Primer | 5’- GTCTAGGACGCAGCAGATTG-3’ |
| Human OCLN Reverse Primer | 5’- TGGACTTTCAAGAGGCCTGG-3’ |
| Human CLDN1 Forward Primer | 5’- CCCAGTCAATGCCAGGTACG-3’ |
| Human CLDN1 Reverse Primer | 5’- ACACGTAGTCTTTCCCGCTG-3’ |
| Human CLDN2 Forward Primer | 5’- CCTTTATCACCTCAGCCCGT-3’ |
| Human CLDN2 Reverse Primer | 5’- GCATCTAGAAGACAGGGCAGT-3’ |
| **miRNA** |  |
| miR-429 Forward Primer  miR-429 Reverse Primer  U6 Forward Primer  U6 Reverse Primer | 5'- CGACCCGACTAATACTGTCTGG-3'  5'- TATGCTTGTTCTCGTCTCTGTGTC-3'  5'- TATGCTTGTTCTCGTCTCTGTGTC-3'  5'- CTCGCTTCGGCAGCACA-3' |
