# Supplementary material for: EPDR1, Which Is Negatively Regulated by miR-429, Suppresses Epithelial Ovarian Cancer Progression via PI3K/AKT Signaling Pathway
Source: Front Oncol. 2021 Dec 23;11:751567. doi: 10.3389/fonc.2021.751567 (PMC8733570; doi:10.3389/fonc.2021.751567)
Supplement: Supplementary file 5 [file Table_4.docx]

| **Supplementary TableS4. Identified miRNAs from database** | |
| --- | --- |
| **Starbase** | **GSE47841** |
| hsa-miR-17-5p | hsa-miR-200a* |
| hsa-miR-18a-5p | hsa-miR-200c |
| hsa-miR-19a-3p | hsa-miR-141 |
| hsa-miR-19b-3p | hsa-miR-200b* |
| hsa-miR-20a-5p | hsa-miR-200a |
| hsa-miR-21-5p | hsa-miR-200b |
| hsa-miR-23a-3p | hsa-miR-106a |
| hsa-miR-24-3p | hsa-miR-182 |
| hsa-miR-25-3p | hsa-miR-425 |
| hsa-miR-27a-3p | hsa-miR-17 |
| hsa-miR-28-5p | hsa-miR-93 |
| hsa-miR-30a-5p | hsa-miR-18a |
| hsa-miR-31-5p | hsa-miR-296-3p |
| hsa-miR-32-5p | hsa-miR-93* |
| hsa-miR-33a-5p | hsa-miR-200c* |
| hsa-miR-92a-3p | hsa-miR-18b |
| hsa-miR-93-5p | hsa-miR-20a |
| hsa-miR-96-5p | hsa-miR-378* |
| hsa-miR-99a-5p | hsa-miR-15b |
| hsa-miR-100-5p | hsa-miR-106b |
| hsa-miR-101-3p | hsa-miR-664 |
| hsa-miR-103a-3p | hsa-miR-4298 |
| hsa-miR-106a-5p | hsa-miR-183 |
| hsa-miR-107 | hsa-miR-24-2* |
| hsa-miR-192-5p | hsa-miR-106b* |
| hsa-miR-199a-5p | hsa-miR-27a |
| hsa-miR-199a-3p | hsa-miR-18a* |
| hsa-miR-148a-3p | hsa-miR-203 |
| hsa-miR-30c-5p | hsa-miR-378c |
| hsa-miR-30d-5p  hsa-miR-10a-5p  hsa-miR-10b-5p  hsa-miR-181a-5p  hsa-miR-181b-5p  hsa-miR-181c-5p  hsa-miR-182-5p  hsa-miR-183-5p  hsa-miR-199b-5p  hsa-miR-204-5p  hsa-miR-211-5p  hsa-miR-212-3p  hsa-miR-215-5p  hsa-miR-217  hsa-miR-218-5p  hsa-miR-219a-5p  hsa-miR-223-3p  hsa-miR-224-5p  hsa-miR-200b-3p  hsa-miR-23b-3p  hsa-miR-27b-3p  hsa-miR-30b-5p  hsa-miR-122-5p  hsa-miR-124-3p  hsa-miR-125b-5p  hsa-miR-130a-3p  hsa-miR-132-3p  hsa-miR-135a-5p  hsa-miR-137  hsa-miR-142-5p  hsa-miR-143-3p  hsa-miR-144-3p  hsa-miR-145-5p  hsa-miR-152-3p  hsa-miR-153-3p  hsa-miR-125a-5p  hsa-miR-126-3p  hsa-miR-154-5p  hsa-miR-186-5p  hsa-miR-200c-3p  hsa-miR-106b-5p  hsa-miR-302a-3p  hsa-miR-301a-3p  hsa-miR-99b-5p  hsa-miR-130b-3p  hsa-miR-30e-5p  hsa-miR-362-5p  hsa-miR-363-3p  hsa-miR-365a-3p  hsa-miR-302b-3p  hsa-miR-367-3p  hsa-miR-369-3p  hsa-miR-372-3p  hsa-miR-373-3p  hsa-miR-374a-5p  hsa-miR-377-3p  hsa-miR-381-3p  hsa-miR-342-3p  hsa-miR-323a-3p  hsa-miR-135b-5p  hsa-miR-148b-3p  hsa-miR-339-5p  hsa-miR-335-5p  hsa-miR-345-5p  hsa-miR-384  hsa-miR-422a  hsa-miR-18b-5p  hsa-miR-20b-5p  hsa-miR-448  hsa-miR-429  hsa-miR-410-3p  hsa-miR-485-5p  hsa-miR-489-3p  hsa-miR-181d-5p  hsa-miR-512-3p  hsa-miR-520e  hsa-miR-520f-3p  hsa-miR-519c-5p  hsa-miR-520a-3p  hsa-miR-526b-5p  hsa-miR-526b-3p  hsa-miR-526a  hsa-miR-520c-3p  hsa-miR-517a-3p  hsa-miR-590-5p  hsa-miR-651-5p  hsa-miR-411-5p  hsa-miR-656-3p  hsa-miR-421  hsa-miR-542-3p | hsa-miR-1913  hsa-miR-15a  hsa-miR-23a  hsa-miR-23a*  hsa-miR-4317  hsa-miR-205  hsa-miR-625  hsa-miR-187  hsa-miR-378  hsa-miR-3195  hsa-miR-671-3p  hsa-miR-130b  hsa-miR-2277  hsa-miR-135b*  hsa-miR-940  hsa-miR-1307  hsa-miR-422a  hsa-miR-16  hsa-miR-27a*  hsa-miR-421  hsa-miR-181d  hsa-miR-1228  hsa-miR-595  hsa-miR-429  hsa-miR-92a-1*  hsa-miR-425*  hsa-miR-1910  hsa-miR-126  hsa-miR-20b  hsa-miR-301a  hsa-miR-934  hsa-miR-224  hsa-miR-25*  hsa-miR-885-5p  hsa-miR-92b  hsa-miR-138-1*  hsa-miR-221  hsa-miR-1825  hsa-miR-25  hsa-miR-3187  hsa-miR-449b*  hsa-miR-149  hsa-miR-128  hsa-miR-877  hsa-miR-1909*  hsa-miR-183*  hsa-miR-449b  hsa-miR-3178  hsa-miR-449a  hsa-miR-346  hsa-miR-27b*  hsa-miR-30b*  hsa-miR-449c  hsa-miR-210  hsa-miR-1973  hsa-miR-369-3p |
