# Supplementary material for: EPDR1, Which Is Negatively Regulated by miR-429, Suppresses Epithelial Ovarian Cancer Progression via PI3K/AKT Signaling Pathway
Source: Front Oncol. 2021 Dec 23;11:751567. doi: 10.3389/fonc.2021.751567 (PMC8733570; doi:10.3389/fonc.2021.751567)
Supplement: Supplementary file 5 [file Table_3.docx]

frontiers

Supplementary TableS3. Sequences of siRNA, mimics and inhibitor

	sense (5'-3')	antisense (5'-3')
Scramble	UUCUCCGAACGUGUCACGUTT	ACGUGACACGUUCGGAGAATT
EPDR1-si1	CUGCAAGAGAUUAUUUGAATT	UUCAAAUAAUCUCUUGCAGTT
EPDR1-si2	CCUCUUGACAUUCCUCAAATT	UUUGAGGAAUGUCAAGAGGTT
Control-miR	UGGUUUACAUGUCGACUAA	UGGUUUACAUGUUGUGUGA
miR-429	UAAUACUGUCUGGUAAAACCGU	GGUUUUACCAGACAGUAUUAUU
miR-421	AUCAACAGACAUUAAUUGGGCGC	GCGCCCAAUUAAUGUCUGUUGAU
miR-422a	ACUGGACUUAGGGUCAGAAGGC	CUUCUGACCCUAAGUCCAGUUU
Control anti-miR	UGGUUUACAUGUUUUCUGA	UGGUUUACAUGUUUUCCUA
anti-miR-429	ACGGUUUUACCAGACAGUAUUA	
